# Impact of glucose-to-lymphocyte ratio on mortality in patients with pneumonia: A retrospective cohort study based on MIMIC-IV and eICU-CRD

**DOI:** 10.1371/journal.pone.0338579

**Published:** 2026-01-09

**Authors:** Mengjiao Xu, Yikun Guo, Jun Yan, Lei Li, Linyang Wang

**Affiliations:** 1 China Academy of Chinese Medical Sciences, Beijing, China; 2 Dongzhimen Hospital, Beijing University of Chinese Medicine, Beijing, China; Tianjin Normal University, CHINA

## Abstract

**Background:**

This study aimed to explore the nonlinear positive correlation between the glucose-to-lymphocyte ratio (GLR) and increased risk of in-hospital mortality and ICU mortality in critically ill patients with pneumonia.

**Methods:**

This was a retrospective observational cohort study and data were obtained from the Medical Information Mart for Intensive Care-IV (MIMIC IV) database and eICU-Collaborative Research Database (eICU-CRD) database. The primary outcome was in-hospital mortality and ICU mortality.According to the GLR, the participants were divided into quartiles (Q1–Q4).Kaplan–Meier analysis was used to compare the mortality of the above four groups.Multivariate Cox regression analysis and restricted cubic spline regression was used to evaluate the association between GLR and in-hospital mortality and ICU mortality in patients with pneumonia. In addition, the data of patients with bacterial pneumonia were extracted using MIMIC-IV database, and Kaplan-Meier analysis and Cox regression analysis were also used.

**Results:**

1,961 patients from the MIMIC-IV cohort were included. Logistics regression analysis showed that an elevated GLR was significantly associated with all-cause mortality. After adjusting for confounding factors, patients with an elevated GLR showed a significant correlation with in-hospital mortality [HR (95% CI): 1.84 (1.36–2.51), p < 0.001] and ICU mortality [HR (95% CI): 1.47 (1.04–2.17), p = 0.049]. Kaplan-Meier survival analysis curves indicated that patients with higher GLR levels had significantly lower survival probabilities. The restricted cubic spline regression model revealed that a nonlinear positive correlation between GLR levels and increased risk of death among patients with pneumonia.6,405 patients from the eICU-CRD cohort were included. Logistics regression analysis showed that, after adjusting for confounding factors, patients with an elevated GLR had a significant association with in-hospital mortality [HR (95% CI): 1.18 (1.00–1.38), p = 0.047]. The Kaplan-Meier survival analysis curves indicated that patients with higher GLR levels had significantly lower survival probabilities. The restricted cubic spline regression model revealed that a nonlinear positive correlation between GLR levels and increased risk of death among patients with pneumonia.Furthermore, data extracted from the MIMIC-IV cohort demonstrated that the predictive performance of GLR for all-cause mortality remained robust among patients with bacterial pneumonia.

**Conclusions:**

GLR is significantly associated with increased all-cause mortality in patients with pneumonia. This finding suggests that GLR may help identify people with pneumonia at high risk of mortality.

## 1. Introduction

Severe pneumonia is an acute and critical respiratory disease, and its morbidity and mortality have been a major challenge in the medical field. Despite rapid advances in intensive care medicine, severe pneumonia remains one of the leading causes of death in the intensive care unit (ICU) [1].According to a U.S. report, more than 1.5 million adults are hospitalized each year for community-acquired pneumonia (CAP);Mortality rate during hospitalization reaches 6.5%, and about 1/3 of CAP inpatients die within 1 year [2].Pneumonia is one of the major infectious diseases causing deaths of all ages worldwide, and its global impact has become undeniable, posing a severe public health challenge [3,4].According to the 2019 Global Burden of Diseases (GBD) study, children <5 years old and adults >70 years old are the groups most affected by pneumonia [3,5].Severe pneumonia causes damage to the lungs and the entire body. As the condition worsens, patients may develop various complications such as respiratory failure, septic shock, and multiple organ dysfunction syndrome [6]. Even after recovery, patients may still suffer from long-term sequelae,which not only aggravates the healthcare burden, but also causes tremendous socioeconomic pressure [7].Therefore, identification of risk factors associated with adverse outcomes in patients with pneumonia is essential to optimize treatment strategies, prevent deterioration, and improve patient prognosis. Previous studies have shown that clinical biomarkers such as red blood cell distribution width and arterial blood lactate to serum albumin ratio (LAR) correlate with pneumonia prognosis [8,9].Nevertheless, a widely accepted biomarker that can predict the prognosis of patients with severe pneumonia has not yet been established. Therefore, it is particularly important to explore novel biomarkers and determine more precise risk stratification for early and accurate assessment of the prognosis of critically ill patients with pneumonia.

Glucose-to-lymphocyte ratio (GLR) is a novel prognostic biomarker discovered in recent years. Compared with traditional biomarkers, GLR combines information from both blood glucose levels and lymphocyte counts to provide a more comprehensive view of a patient’s metabolic and immune status, and an elevated GLR implies a disturbance in glucose metabolism and an imbalance in immune function [10].Current research indicates that GLR has been utilized in assessing the prognosis of various critical illnesses, including acute exacerbation of chronic obstructive pulmonary disease [11], acute respiratory distress syndrome [12], non-traumatic cerebral hemorrhage [13], and acute pancreatitis [14], and has demonstrated a good ability to predict mortality. For example, it has been noted that GLR independently predicts 14-day in-hospital mortality in patients with acute myocardial infarction admitted to the ICU and has superior predictive value compared to using glucose levels or lymphocyte counts as predictors alone [15].Although the predictive ability of GLR has been widely validated in a wide range of acute and critical conditions, its value in predicting mortality in patients with pneumonia has not yet been demonstrated. Therefore, our study focused on exploring the predictive ability of GLR for ICU mortality and in-hospital mortality in patients with pneumonia, with the aim of being able to provide new and valuable references for the early identification and effective intervention of critically ill patients with pneumonia in clinical practice.

## 2. Methods

### 2.1. Study participants

In this study, clinical data related to pneumonia patients were obtained from Medical Information Mart for Intensive Care-IV (MIMIC-IV) database and eICU-Collaborative Research Database(eICU-CRD) for retrospective cohort study. MIMIC-IV is an open access database that includes data on patients admitted to the ICU at Beth Israel Deaconess Medical Center between 2008 and 2019 [16].The data in the eICU-CRD encompasses the hospitalization information of patients admitted to multiple Intensive Care Units (ICUs) across the United States in 2014 and 2015 [17].One of the authors, Yiku Guo, passed the Cooperative Institution Training Program (CITI) and was granted access to the databases, and collected clinical information from both databases.To ensure the confidentiality of patient data, all personal information has been anonymized by replacing patient identities with random codes, eliminating the need for patient consent and ethical approval. As this study is a secondary analysis of the MIMIC-IV and eICU-CRD, ethical approval and related documents are not required.

We extracted patients diagnosed with pneumonia within 24 hours of admission from the MIMIC-IV database and eICU-CRD for our study. The exclusion criteria were as follows: (1) age < 18 years or age > 100 years; (2) ICU stay <48 hours; (3).Lack of lymphocyte and glucose data within 24 hours

**Fig 1:**
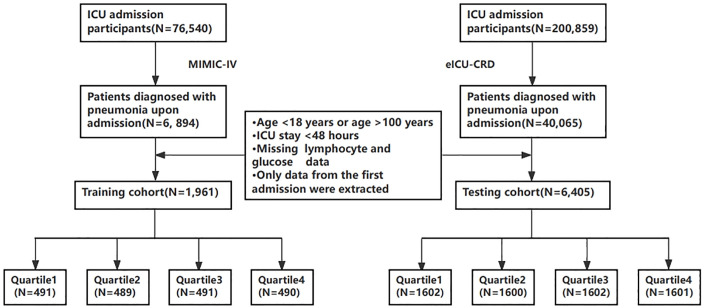
Flowchart of patient selection.

A total of 8,062 patients who met the inclusion and exclusion criteria were included in this study, including 1,961 patients in the MIMIC-IV cohort and 6,405 patients in the eICU-CRD cohort.The flowchart for patients screening is presented in Fig 1.

### 2.2. Data extraction

Structured Query Language (SQL) was utilized in Navigate Premium (version 16) software to extract relevant data from patients with pneumonia from MIMIC-IV and eIUC-CRD, covering variables such as demographics, vital signs, Laboratory parameters, comorbidities, scoring systems, and treatment measures. Furthermore, to ensure the reliability of the analysis, variables with missing values exceeding 20% were excluded, while multiple imputation was performed for variables with missing values below 20%.Data with missing values exceeding 20% have been listed in Supporting information: S1.

### 2.3. Clinical outcomes

The primary outcomes were in-hospital mortality and ICU mortality in patients with pneumonia.

### 2.4. Statistical analysis

The Kolmogorov-Smirnov test was used to evaluate the normal distribution of continuous variables. Continuous variables with a normal distribution were described by means ± standard deviation (mean ± SD) and compared using t-tests. Continuous variables with non-normal distributions were represented as median (interquartile range) [M (IQR)] and compared using the Wilcoxon rank-sum test. Categorical variables presented as numbers and percentages(%) and compared using Chi-square test or Fisher’s exact test.

Kaplan-Meier survival analysis was employed to evaluate the incidence rate of major outcome events across distinct stratified groups based on GLR.Cox proportional hazards models were used to calculate the hazard ratio (HR) and 95% confidence interval (CI) between GLR and mortality, and adjusted for multiple variables. A restricted cubic spline (RCS) model was used to investigate the potential dose–response association between GLR and all-cause mortality in patients with pneumonia.In RCS, 4 knots have been selected to ensure the flexibility of the model while avoiding overfitting. The knot positions are set based on the quantiles of the data. In this case, the quartiles are chosen as the knot positions, which can effectively capture the changing trends in the data.Finally, subgroup analysis was used to investigate the consistency of the prognostic value of GLR within different subgroups. These subgroups were based on age; sex; complications, such as hypertension, diabetes mellitus, heart failure, myocardial infarction, cancer, stroke, COPD, and atrial fibrillation; and interventions, such as mechanical ventilation, vasoactive drugs.All analyses mandated a significance threshold of *P* < 0.05 (two-tailed) and were executed via R software (version 4.2.2)

## 3. Results

### 3.1. Baseline demographic and clinical characteristics

A total of 1961 patients with pneumonia were enrolled in MIMIC-IV cohort (mean age [SD]: 65.8 ± 17 years; 42.2% female,57.8% male);the in-hospital,and ICU mortality were 21.3%, 14% respectively.A total of 6405 patients with pneumonia were enrolled in eICU-CRD cohort (mean age[SD]:66.6 ± 15.8 years;45.9% female,54.1%male);the in-hospital,and ICU mortality were 16.4%, 10.2% respectively. We divided patients into 4 groups based on their GLR level.

MIMIC-IV cohort showed, compared with other groups (Q1-Q3), patients with higher GLR levels (Q4) usually have higher RR, APS III, WBC, RDW, Glu, BUN, Cr, and lower RBC, lymphocyte, PLT, HGB, Na, and PO2.The prevalence of diabetes, COPD and atrial fibrillation was higher.Details of the baseline information of MIMIC-IV cohort are listed in Table 1.

**Table 1:** Comparison of baseline features between groups stratified by GLR quartile of MIMIC-IV cohort.

Categories	Q1 (N = 491)	Q2(N = 489)	Q3 (N = 491)	Q4 (N = 490)	Total (N = 1961)	P-value
Demography						
Gender, n (%)						0.155
Female	225 (45.8%)	209 (42.7%)	190 (38.7%)	204 (41.6%)	828 (42.2%)	
Male	266 (54.2%)	280 (57.3%)	301 (61.3%)	286 (58.4%)	1133 (57.8%)	
Age (year)	61.9(42.5,81.1)	65.7(49,82.4)	67.4(51.3,83.5)	68.5(53.9,83.1)	65.8(48.8,82.8)	<0.001
Weight (kg)	82.6(55.6,109.6)	82.5(56.9,108.1)	82.9(58.7,107.1)	81(56,106)	82.2(56.7,107.7)	0.642
Vital signs						
HR (times/min)	91.7(71.4,112)	94.9(73.2,116.6)	93.1(70,116.2)	94.7(72.2,117.2)	93.6(71.6,115.6)	0.075
RR (times/min)	20.6(13.9,27.3)	21.2(14.6,27.8)	21.1(14.5,27.7)	22(15.5,28.5)	21.2(14.6,27.8)	0.016
Temperature (**°C)**	36.9(35.2,38.6)	37(36.4,37.6)	36.6(35.9,37.3)	36.9(36.2,37.6)	36.8(34.7,38.9)	0.027
SpO_2_ (%)	96.5(92.2,100.8)	96.3(92.1,100.5)	97.8(93.9,101.7)	96(92,100)	96.7(94.7,98.7)	0.489
DBP (mmHg)	120.2(96,144.4)	122.9(97.6,148.2)	120.3(95.8,144.8)	120.8(96.9,144.7)	121.1(96.6,145.6)	0.371
SBP (mmHg)	69.8(51.6,88)	72.5(52.3,92.7)	70.5(50.8,90.2)	69.5(51.5,87.5)	70.6(51.5,89.7)	0.107
MBP (mmHg)	97.1(76.4,117.8)	84.9(64.5,105.3)	83.3(64.1,102.5)	82.5(64.7,100.3)	87(66.2,107.8)	0.475
Comorbidities, n (%)						
Hypertension	166 (33.8%)	165 (33.7%)	163 (33.2%)	174 (35.5%)	668 (34.1%)	0.883
Diabetes	101 (20.6%)	121 (24.7%)	172 (35%)	185 (37.8%)	579 (29.5%)	<0.001
Heart Failure	151 (30.8%)	166 (33.9%)	178 (36.3%)	177 (36.1%)	672 (34.3%)	0.229
Myocardial Infarction	67 (13.6%)	78 (16%)	79 (16.1%)	96 (19.6%)	320 (16.3%)	0.09
Cancer	60 (12.2%)	70 (14.3%)	86 (17.5%)	89 (18.2%)	305 (15.6%)	0.033
Stroke	26 (5.3%)	35 (7.2%)	33 (6.7%)	40 (8.2%)	134 (6.8%)	0.351
COPD	75 (15.3%)	82 (16.8%)	90 (18.3%)	114 (23.3%)	361 (18.4%)	0.008
Atrial Fibrillation	152 (31%)	156 (31.9%)	183 (37.3%)	185 (37.8%)	676 (34.5%)	0.043
Scores system						
APS III	47.8(27.2,68.4)	49.4(28.8,70)	52.6(31.4,73.8)	56.6(35.8,77.4)	51.6(30.4,72.8)	<0.001
GCS	13.4(10.3,16.5)	13.4(10.6,16.2)	13.4(10.4,16.4)	13.6(10.7,16.5)	13.4(10.4,16.4)	0.688
Laboratory tests						
WBC (K/UL)	11.8(4.6,19)	13.6(5.5,21.7)	14.1(6.4,21.8)	16.4(8.9,23.9)	13.9(6.8,21)	<0.001
RBC (m/uL)	3.5(2.7,4.3)	3.6(2.8,4.4)	3.5(2.6, 4.4)	3.4(2.6,5)	3.5(2.7,4.3)	0.037
Neutrophils (K/UL)	11.7(3.4,20)	11.7(3.9,19.5)	11.5(3.8,19.2)	10.4(3.3,17.5)	11.3 ± 7.8	0.014
lymphocyte (K/UL)	1.(0.6,1.8)	1.3(0.8,1.8)	0.9(0.5,1.3)	0.4(0.1,0.7)	0.6(0.4,0.8)	<0.001
PLT (K/μL)	210.3(95.2,325.4)	204.7(92.8,316.6)	199.7(91.9,307.5)	178.3(70.8,285.8)	198.2(87,309.4)	<0.001
HGB (g/dL)	10.5(8.2,12.8)	10.7(8.3,13.1)	10.4(7.9,12.9)	10.2(7.8,12.6)	10.4(8,12.8)	0.017
RDW (K/μL)	15.3(12.7,17.9)	15.3(12.8,17.8)	15.6(12.9,18.3)	15.7(13,18.4)	15.5(12.9,18.1)	0.022
Na (mmol/L)	139.1(133.4,144.8)	138.7(132,145.4)	138.9(132.6,145.2)	137.6(131.3,143.9)	138.6(132.3,144.9)	<0.001
K (mmol/L)	4.2(3.4,5)	4.2(3.3,5.1)	4.2(3.4,5)	4.3(3.5,5.1)	4.2(3.4,5)	0.113
Ca (mg/dL)	8.3(7.5,9.1)	8.3(7.5,9.1)	8.2(7.4,9)	8.2(7.3,9.1)	8.3(7.5,9.1)	0.098
Glu (K/UL)	113.9(80.6,147.2)	135.7(84.9,186.5)	164.2(90.3,238.1)	199(91.5,306.5)	153.2(74.6,231.8)	<0.001
Ph	7.4(7.3,7.5)	7.4(7.3,7.5)	7.3(7.2,7.4)	7.3(7.2,7.4)	7.4(7.3,7.5)	0.004
PCO2 (mmHg)	43.6(31.1,56.1)	44.9(31.6,58.2)	43.7(30.2,57.2)	43.9(30.8,57)	44(30.9,57.1)	0.414
PO2 (mmHg)	119.7(16.6,222.8)	106.4(22.6,190.2)	106.9(12.8,201)	90.8(17,164.6)	106(16.1,195.9)	<0.001
BUN (mg/dL)	22.9(5.9,39.9)	27.4(2.7,52.1)	31.7(5.5,57.9)	34.9(8.4,61.4)	29.2(4.9,53.5)	<0.001
Cr (mg/dL)	1.3(0.2,2.4)	1.5(0.1,2.9)	1.6(0.2,3)	1.8(0.1,3.5)	1.5(0,3)	<0.001
Treatment						
Mechanical ventilation, n (%)	459 (93.5%)	451 (92.2%)	463 (94.3%)	462 (94.3%)	1835 (93.6%)	0.509
Vasoactive drugs,n (%)	248 (50.5%)	261 (53.4%)	269 (54.8%)	279 (56.9%)	1057 (53.9%)	0.231
Outcome						
In-hospital mortality, n (%)	64 (13%)	93 (19%)	118 (24%)	142 (29%)	417 (21.3%)	<0.001
ICU mortality, n (%)	42 (8.6%)	58 (11.9%)	81 (16.5%)	93 (19%)	274 (14%)	<0.001

HR: Heart Rate; RR: Respiratory Rate; DBP: Diastolic Blood Pressure; SBP: Systolic Blood Pressure; MBP: Mean Blood Pressure; COPD:Chronic Obstructive Pulmonary Disease;APS III: Acute Physiology Score III;

GCS: Glasgow Coma Scale;WBC:White Blood Cell; RBC:Red Blood Cell; PLT:Platelet; HGB: Hemoglobin;RDW: Red Blood Cell Distribution Width; Glu: Glucose; BUN:Blood Urea Nitrogen; Cr:Creatinine

eICU-CRD cohort showed,compared with other groups (Q1-Q3), patients with higher GLR levels (Q4) usually have higher RR, temperature,GCS、WBC、Glu、BUN,and lower SpO_2_、RBC、lymphocyte、PLT、HGB、Na.The prevalence of cancer and atrial fibrillation was higher.Details of the baseline information of eICU-CRD cohort are listed in Supporting information:S2 Table.

### 3.2. All-cause mortality in different groups

**Fig 2:**
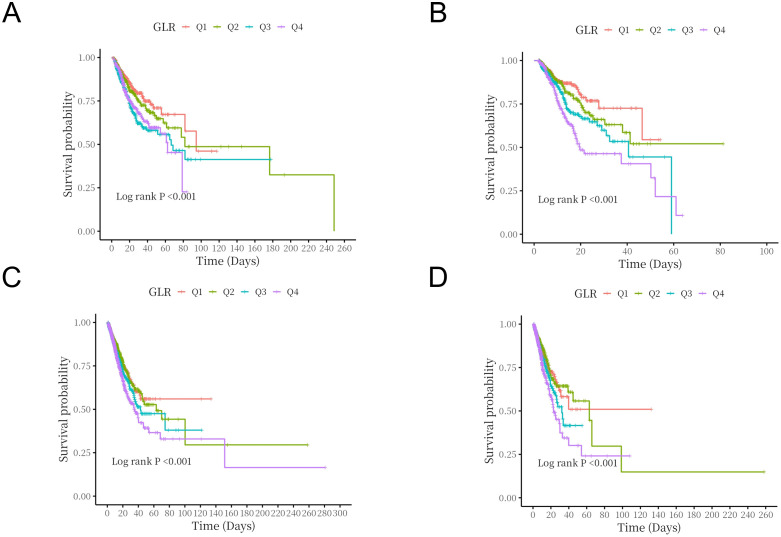
Kaplan-Meier survival analysis curves for all-cause mortality. (A) Kaplan-Meier survival analysis curves for in-hospital mortality of MIMIC-IV cohort; (B) Kaplan-Meier survival analysis curves for ICU mortality of MIMIC-IV cohort; (C) Kaplan-Meier survival analysis curves for in-hospital mortality of eICU-CRD cohort; (D) Kaplan-Meier survival analysis curves for ICU mortality of eICU-CRD cohort.

Fig 2 showed Kaplan-Meier survival analysis curves, which demonstrated both in the MIMIC-IV cohort and the eICU-CRD cohort, there were differences in in-hospital mortality and ICU mortality among the four groups (log-rank *p* < 0.001), and the mortality increased as GLR quartiles increased,with the lowest in-hospital survival and ICU survival in the Q4 group.

### 3.3. Association between all-cause mortality and GLR

Table 2 showed multivariate Cox regression models, which assessed the association between GLR and all-cause mortality among patients with pneumonia in the MIMIC-IV and eICU-CRD cohorts. The results demonstrated that, after adjusting for confounding factors, the higher quartile(Q4) of GLR in the MIMIC-IV cohort emerged as a risk factor for in-hospital mortality among patients with pneumonia in Model I [HR (95% CI): 2.25 (1.68–3.03), p < 0.001], Model II [HR (95% CI): 1.82 (1.35–2.46), p < 0.001], Model III [HR (95% CI): 1.80 (1.33–2.42), p < 0.001], and Model IV [HR (95% CI): 1.84 (1.36–2.51), p < 0.001].The higher quartile(Q4) of GLR was also significantly associated with increased ICU mortality in Model I [HR (95% CI): 2.41 (1.68–3.48), p < 0.001], Model II [HR (95% CI): 1.87 (1.29–2.7), p = 0.001], Model III [HR (95% CI): 1.81 (1.24–2.63), p = 0.002], and Model IV [HR (95% CI): 1.47 (1.04–2.17), p = 0.049], suggesting that compared to subjects in the lowest quartile, the all-cause mortality among patients with pneumonia increased with rising GLR levels. In the eICU-CRD cohort, similar results were observed for in-hospital mortality. However, for ICU mortality, after adjusting for confounding factors, the higher quartile(Q4) of GLR was not significant in Models II-IV, indicating that it was not an independent prognostic factor for ICU mortality.

**Table 2:** Cox proportional hazard ratio for all-cause mortality.

Categories		Model I		Model II		Model III		Model IV	
HR (95%CI)	P	HR (95%CI)	P	HR (95%CI)	P	HR (95%CI)	P
In-hospital mortality (MIMIC-IV)	Q1	Ref.		Ref.		Ref.		Ref.	
	Q2	1.38 (1.01-1.91)	0.046	1.28 (0.93-1.77)	0.128	1.24 (0.90-1.71)	0.188	1.19 (0.86-1.6)	0.289
	Q3	1.75 (1.29-2.37)	<0.001	1.53 (1.13-2.08)	0.006	1.50 (1.11-2.04)	0.009	1.51 (1.10-2.07)	0.01
	Q4	2.25 (1.68-3.03)	<0.001	1.82 (1.35-2.46)	<0.001	1.80 (1.33-2.42)	<0.001	1.84 (1.36-2.51)	<0.001
ICU mortality (MIMIC-IV)	Q1	Ref.	P	Ref.	P	Ref.	P	Ref.	P
	Q2	1.30 (0.88-1.94)	0.192	1.23 (0.83-1.84)	0.302	1.16 (0.78-1.73)	0.476	1.04 (0.69-1.57)	0.84
	Q3	1.74 (1.20-2.53)	0.004	1.61 (1.10-2.34)	0.013	1.63 (1.12-2.37)	0.011	1.39 (0.94-2.03)	0.096
	Q4	2.41 (1.68-3.48)	<0.001	1.87 (1.29-2.7)	0.001	1.81 (1.24-2.63)	0.002	1.47 (1.04-2.17)	0.049
In-hospital mortality (eICU-CRD)	Q1	Ref.	P	Ref.	P	Ref.	P	Ref.	P
	Q2	0.95 (0.79-1.14)	0.568	0.89 (0.74-1.07)	0.211	0.89 (0.74-1.08)	0.233	0.95 (0.79-1.15)	0.604
	Q3	1.17 (0.98-1.40)	0.082	1.11 (0.93-1.33)	0.251	1.10 (0.92-1.32)	0.291	1.14 (0.94-1.37)	0.174
	Q4	1.33 (1.12-1.58)	0.001	1.20 (1.01-1.4)	0.042	1.19 (1.01-1.40)	0.044	1.18 (1.00-1.38)	0.047
ICU mortality (eICU-CRD)	Q1	Ref.	P	Ref.	P	Ref.	P	Ref.	P
	Q2	0.89 (0.70-1.13)	0.337	0.80 (0.63-1.01)	0.065	0.81 (0.64-1.03)	0.091	0.86 (0.67-1.10)	0.227
	Q3	1.17 (0.94-1.46)	0.169	1.10 (0.88-1.37)	0.423	1.11 (0.88-1.39)	0.372	1.11 (0.88-1.41)	0.374
	Q4	1.38 (1.11-1.71)	0.004	1.15 (0.92-1.43)	0.224	1.14 (0.91-1.42)	0.245	1.15 (0.89-1.47)	0.281

**Model 1** was unadjusted;

**Model 2** was adjusted for age, sex, weight, heart rate, respiratory rate, temperature, MBP, SBP, DBP, SpO2, APSIII, GCS;**Model 3** was adjusted for age, sex, weight, heart rate, respiratory rate, temperature, MBP, SBP, DBP, SpO2, APSIII, GCS, hypertension, diabetes, myocardial infarction, COPD, cancer, atrial fibrillation, liver disease, heart failure; **Model 4** was adjusted for age, sex, weight, heart rate, respiratory rate, temperature, MBP, SBP, DBP, SpO2, APSIII, GCS, hypertension, diabetes, myocardial infarction, COPD, cancer, atrial fibrillation, liver disease, heart failure,WBC、RBC、Neutrophils、lymphocyte、PLT、HGB、RDW、Cl、K、Na、Ca、Glu、AG、Cr、BUN、APTT.

C-index:MIMIC-IV:ICU mortality: 0.713 (0.658–0.738);In-hospital mortality: 0.736 (0.707–0.769);.

eICU-CRD:ICU mortality: 0.722(0.710–0.733);In-hospital mortality: 0.745 (0.711–0.784).

**Fig 3:**
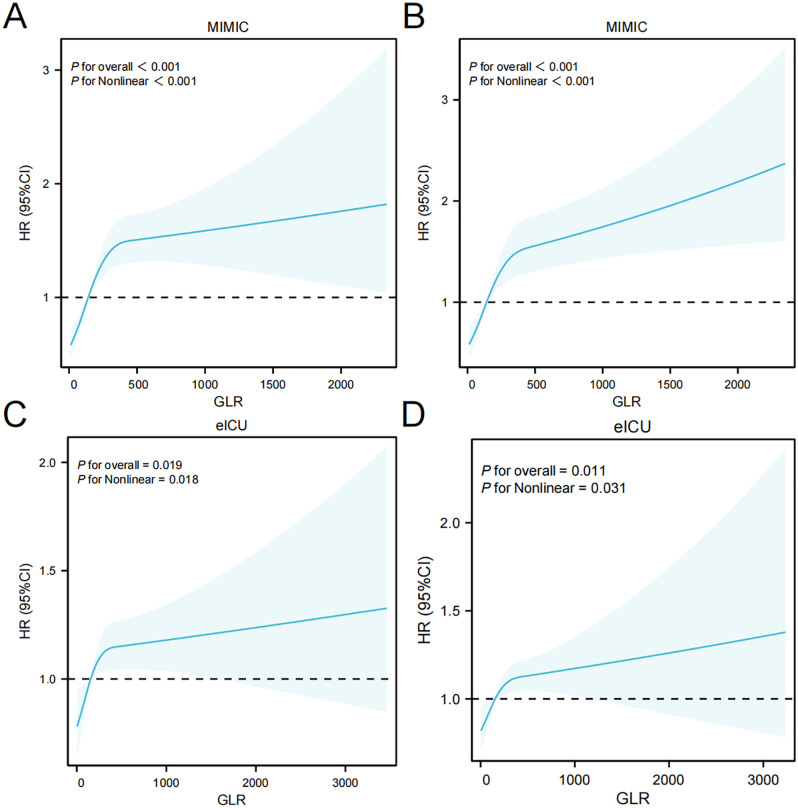
Restricted Cubic Spline of GLR with all-cause mortality. (A) Restricted Cubic Spline for in-hospital mortality of MIMIC-IV cohort;(B) Restricted Cubic Spline for ICU mortality of MIMIC-IV cohort;(C) Restricted Cubic Spline for in-hospital mortality of eICU-CRD cohort;(D) Restricted Cubic Spline for ICU mortality of eICU-CRD cohort.

Fig 3 showed the restricted cubic splines regression model,which demonstrated both in the MIMIC-IV cohort and the eICU-CRD cohort, there was a nonlinear positive correlation trend between GLR and in-hospital mortality as well as ICU mortality (nonlinear P < 0.05). As the GLR level continued to increase, the risk of death for patients also rose.

### 3.4. Subgroup analysis

In order to further analyze the prognostic impact of GLR levels on specific populations, we evaluated the risk stratification values of GLR for the primary outcome in different subgroups of pneumonia patients,such as age, sex, hypertension, diabetes mellitus, heart failure, myocardial infarction, cancer, stroke, COPD, and atrial fibrillation, mechanical ventilation, CRRT(Continuous Renal Replacement Therapy), vasoactive drugs. The data showed that GLR was robust in predicting in-hospital mortality and ICU mortality.

**Fig 4:**
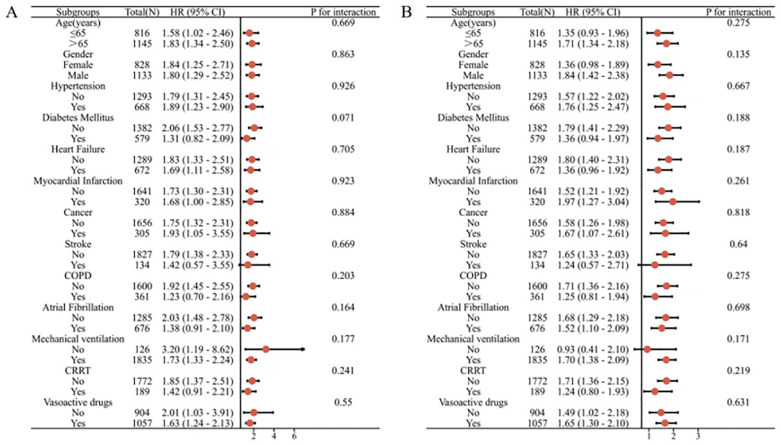
Subgroup analysis of the association between all-cause mortality and GLR. (A)GLR with in-hospital mortality of MIMIC-IV cohort; (B)GLR with ICU mortality of MIMIC-IV cohort.

**Fig 5:**
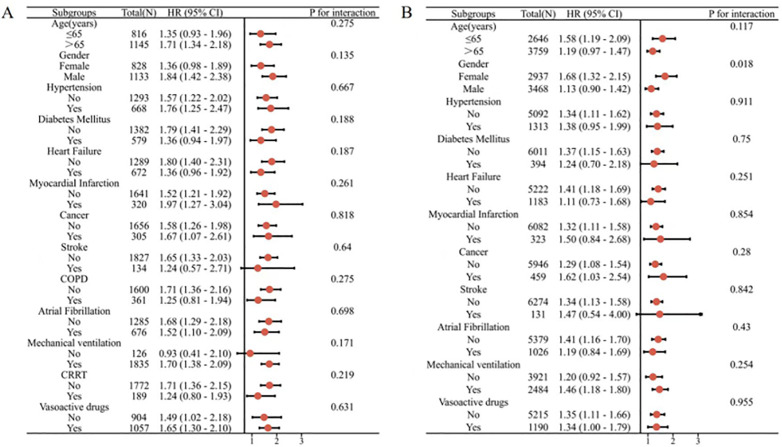
Subgroup analysis of the association between all-cause mortality and GLR. (A)GLR with in-hospital mortality of eICU-CRD cohort;(B)GLR with ICU mortality of eICU-CRD cohort.

The MIMIC-IV cohort (Fig 4) showed no significant interaction of GLR with each subgroup in terms of in-hospital mortality(P for interaction:0.071–0.926) and ICU mortality(P for interaction: 0.135–0.818). The eICU-CRD cohort (Fig 5) showed no significant interaction of GLR with each subgroup in terms of in-hospital mortality(P for interaction: 0.219–0.924).The eICU-CRD cohort (Fig 5) showed no significant interaction of GLR with each subgroup in terms of ICU mortality (P for interaction: 0.117–0.955)except gender; GLR appeared to have more prominent predictive value in female patients with pneumonia[HR (95%CI)1.68 (1.32–2.15), P for interaction = 0.018].

## 4. Bacterial pneumonia

**Fig 6:**
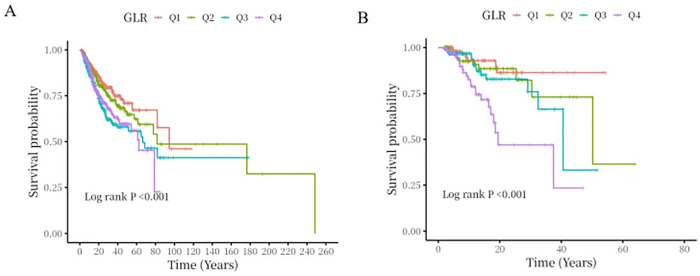
Kaplan-Meier survival analysis curves for all-cause mortality in patients with bacterial pneumonia. (A) Kaplan-Meier survival analysis curves for in-hospital mortality; (B) Kaplan-Meier survival analysis curves for ICU mortality.

We further extracted the relevant data of bacterial pneumonia from the MIMIC-IV database to verify the relationship between GLR and all-cause mortality in patients with bacterial pneumonia. Baseline data table results (Table 3) showed that GLR was significantly associated with in-hospital mortality and ICU mortality. The results of the Kaplan-Meier survival analysis curves(Fig 6) showed that with the increase of GLR level, the prognostic survival probability of patients with bacterial pneumonia continued to decline, and higher GLR(Q4) had the lowest in-hospital survival and ICU survival. Multivariate Cox regression analysis (Details information were listed in Supporting information: S3 Table) showed that, after adjusting for confounders, higher GLR(Q4) was an independent risk factor for pneumonia mortality in all four models.

**Table 3:** The difference of all-cause mortality in patients with bacterial pneumonia under different quartile stratification.

Categories	Q1(N = 149)	Q2(N = 115)	Q3(N = 133)	Q4(N = 117)	total (N = 514)	p
In-hospital mortality	14 (9.4%)	16 (13.9%)	28 (21.1%)	34 (29.1%)	92 (17.9%)	<0.001
ICU mortality	7 (4.7%)	11 (9.6%)	15 (11.3%)	23 (19.7%)	56 (10.9%)	0.002

## 5. Discussion

To our knowledge, this study is the first to investigate the association between GLR and all-cause mortality in patients with pneumonia. GLR was significantly associated with in-hospital mortality and ICU mortality in both MIMIC-IV and eICU-CRD cohorts.After adjusting for multiple confounders, the association remained robust. In the eICU-CRD cohort, although the association between high GLR and increased in-hospital mortality remained strong, the association between GLR and ICU mortality was not significant. After analyzing this phenomenon, we believe that there may be several reasons for it: firstly, the improvement of the patient’s ICU condition after being transferred out of the ICU reduces the ICU mortality, while the in-hospital mortality is not affected. Secondly, in survival analysis, in-hospital mortality and ICU mortality can be considered as competing risks. If a patient dies during hospitalization due to other complications or diseases, it may mask the impact of ICU treatment on their survival rate. Thirdly, ICU patients may need to be transferred to other departments for further treatment due to their condition, which may indirectly affect in-hospital mortality.We further explored the predictive performance of GLR for all-cause mortality of bacterial pneumonia using MIMIC-IV cohort, which still showed good predictive performance of GLR.

As a clinically accessible biomarker, GLR is composed of glucose and lymphocytes, and has shown excellent predictive performance in recent years in the prognosis evaluation of cancers, inflammatory diseases, and respiratory diseases. Multiple clinical studies have verified the important value of GLR in assessing the prognosis of respiratory diseases.Yang et al. reported that GLR and modified Glasgow Prognosis Score (mGPS) are independent prognostic factors for Non-small cell lung cancer (NSCLC) patients, and are negatively correlated with overall survival (OS) of patients with NSCLC [18]. Hu et al. revealed the predictive value of GLR in AECOPD patients and found that GLR can effectively predict the in-hospital mortality of patients with AECOPD admitted to the ICU [11].Subsequently, Zhang et al. further confirmed GLR as an independent risk factor for AECOPD by developing a new predictive model [19].Another study involving 1085 subjects showed that GLR is an independent predictor of in-hospital mortality in patients with acute respiratory distress syndrome(ARDS),with elevated GLR associated with higher mortality [12].These studies suggest that GLR has the potential to emerge as a novel biomarker for predicting respiratory diseases, providing important reference for risk assessment and timely intervention in critically ill patients.

The exact mechanism underlying the association between GLR levels and mortality risk in patients with pneumonia remains unclear. The persistent hyperglycemia under the stress state caused by severe pneumonia has become an important inducing factor for difficult-to-control infection, organ and tissue damage, and disease deterioration.In the complex environment of acute stress and inflammation, the body releases a large amount of cortisol, which inhibits glucose uptake by antagonizing insulin.There is growing evidence that stress hyperglycemia is closely related to poor prognosis in critically ill patients, and this correlation is independent of whether the patient has diabetes or not [20,21].A retrospective study found that regardless of whether patients have a history of diabetes, stress hyperglycemia at admission can exacerbate the progression of COVID-19 imaging, leading to disease deterioration [22].Furthermore, animal experiments have also revealed the potential harm of stress hyperglycemia. Gill S.K. et al. found that stress hyperglycemia can increase the airway bacterial load in normal mice, further emphasizing the importance of controlling blood glucose levels to treat pneumonia [23].

In addition to transient stress hyperglycemia, chronic hyperglycemia is also an important risk factor for pneumonia.For a long time, diabetes, as a common complication of respiratory diseases, has attracted much attention for its close connection with pneumonia.When people with diabetes face stress such as pneumonia, their blood sugar levels tend to fluctuate more sharply, which undoubtedly aggravates the complexity and difficulty of treatment.Studies have shown that people with diabetes have a significantly higher risk of respiratory infection than those without diabetes, and are more likely to develop severe disease [24,25].Diabetes is an important risk factor for pneumonia [26,27]. A population-based case-control study further confirmed that both type 1 and type 2 diabetes are risk factors for hospitalization in patients with pneumonia [26].Chronic hyperglycemia status not only increases the incidence of CAP, aggravates the disease, and increases the incidence of complications and the risk of death [28–30].

As effector cells, lymphocytes play an important role in the complex process of systemic inflammation in critical patients [31].Research shows that severe inflammatory response caused by infection will sharply accelerate the apoptosis of lymphocytes, leading to a decrease in circulating lymphocytes and inducing harmful immunosuppression [32].A study has shown that mice with co-infection of postinfluenza A virus-streptococcus pneumoniae(IAV-SP) exhibit a more significant reduction in lymphocyte subsets compared to mice infected with IAV alone.Another study has shown that pneumonia caused by both Gram-positive and Gram-negative bacteria widely triggers systemic lymphocyte apoptosis in mice [33].These studies suggest that both bacterial and viral infections can lead to a decrease in the number of lymphocytes, which may impair the body’s immune response and affect the course and outcome of the disease.

As the core component of the immune system, lymphocytes not only actively participate in inflammatory reactions but also play a crucial role in immune surveillance. A decrease in their number directly reflects impaired immune function. Immune cell depletion and dysfunction are recognized as key factors affecting the risk of secondary infections and poor prognosis in critically ill patients [34].Diabetics commonly exhibit immune dysfunction, which may be related to lymphocyte depletion, thereby increasing their risk of infection [35,36]. Chronic hyperglycemia triggers changes in the immune system, such as suppression of neutrophils and macrophages, as well as dysfunction of lymphocytes, which collectively constitute the main mechanisms of immune decline in diabetics [35].Diabetes impairs NK cell activity, alters normal T cell differentiation, and damages B cell function [37].This series of immunosuppressive effects is particularly significant in pneumonia patients with diabetes. As a comprehensive indicator, GLR can reflect patients’ blood glucose metabolism and immune status during the disease process, providing a scientific basis for the development of more precise and effective treatment plans.

This study shows that pneumonia patients with elevated GLR have a higher proportion of comorbidities such as COPD, myocardial infarction, and malignant tumors. The relationship between GLR and these diseases is worth investigating. Notably, several studies have validated the predictive value of GLR for the prognosis of these diseases.Hu et al. found that as an easily accessible biomarker, GLR can independently predict in-hospital mortality among AECOPD patients admitted to the ICU, and ROC analysis indicated that GLR had superior predictive ability compared to inflammatory markers such as NLR and PLR [11].Liu et al. discovered that in predicting 14-day in-hospital mortality among patients with acute myocardial infarction, GLR had superior predictive value compared to glucose and lymphocytes alone [15]. Zhang et al.‘s study showed that the preoperative blood glucose to lymphocyte ratio was an independent biomarker for predicting overall survival in patients undergoing pancreatic ductal adenocarcinoma resection [38]. Another study found that in patients with hepatocellular carcinoma treated with sorafenib, GLR was identified as an independent prognostic factor for both progression-free and overall survival [39].

Our study is unique.. Notably, in the initial inclusion and exclusion criteria, we did not exclude diabetic patients, and in subsequent subgroup analyses, we further explored the applicability of GLR in predicting all-cause mortality in pneumonia patients with and without diabetes.According to existing literature, the mechanisms of lymphocytopenia caused by pneumonia alone and that caused by pneumonia complicated with diabetes differ [35,40].Subgroup analysis found no significant interaction between GLR and the presence or absence of diabetes, suggesting that GLR consistently predicts mortality regardless of whether the pneumonia patient has diabetes. This indicates that the effectiveness of GLR as a prognostic indicator is not affected by diabetic status. Additionally, we further explored the predictive performance of GLR for all-cause mortality in bacterial pneumonia. It is generally believed that changes in lymphocytes are more pronounced in viral infections, while in bacterial pneumonia, the number and function of lymphocytes are less affected. Initially, we had reservations about the efficacy of GLR in predicting mortality in bacterial pneumonia, fearing its performance might be weakened. However, further analysis revealed that GLR still demonstrated its significant value as a predictor of all-cause mortality in pneumonia. This may be due to the fact that GLR incorporates information from both blood glucose and lymphocyte counts, with hyperglycemia being more prevalent and significant in bacterial pneumonia, thereby significantly contributing to the predictive value of GLR.

## 6. Advantages and limitation

The strengths of this study lie in its pioneering revelation that GLR is an independent risk factor for all-cause mortality in critically ill pneumonia patients. We employed a dual-database cross-validation approach to effectively expand the sample size. Additionally, subgroup analyses were conducted on patients with and without diabetes to separately evaluate GLR’s predictive value for all-cause mortality, offering more precise evidence for clinical decision-making across diverse disease backgrounds. Furthermore, we exclusively assessed GLR’s predictive performance in bacterial pneumonia patients, aligning our findings more closely with clinical practice.However, limitations exist. First, only initial GLR at admission was used, preventing assessment of this index on disease prognosis over time.Second, the database’s time scope restricted access to ICU microbiological data from the past five years. Third, lacking necessary data, we couldn’t compare GLR’s predictive value with CRP, PCT, or NLR. Fourth, pneumonia diagnoses relied on administrative coding, potentially introducing misclassification, and treatment information varied across databases, hindering precise regimen capture. Fifth, focusing on critically ill patients led to potential selection bias, limiting generalizability. Sixth, viral or fungal pneumonia analyses were absent.This may obscure pathogen-specific effects, thereby limiting the generalizability of the results.Future prospective studies should validate GLR’s predictive value across pneumonia types.Despite these limitations, rigorous statistical methods and subgroup analyses enhanced result validity. Given its clinical significance, we’ve prioritized this topic for future research and emphasize the urgency of high-quality prospective studies. Improvements will include adopting standardized diagnostic criteria to minimize misclassification and recording detailed treatment regimens for comprehensive analysis. Our research will focus on exploring GLR’s dynamic impact on prognosis and comparing its predictive value with other indicators. Moreover, we’ll expand our scope to outpatient and general ward patients to reduce bias and conduct specific studies on viral and fungal pneumonia to uncover their unique clinical features.

## 7. Conclusions

GLR is closely associated with in-hospital mortality and ICU mortality in patients with pneumonia, and can be used as an important risk predictor of prognosis in critically ill patients with pneumonia, and its effectiveness is not affected by diabetes status. GLR’s good prediction of all-cause mortality also applies to bacterial pneumonia.

## Supporting information

S1 Table Variables with missing values exceeding 20%. (XLS)

S2 Table Comparison of baseline features between groups stratified by GLR quartile of eICU-CRD cohort. (PDF)

S3 Table Cox proportional hazard ratio for all-cause mortality in patients with bacterial pneumonia. (PDF)

## References

[1] Huang L, Zhang X, Pang L, Sheng P, Wang Y, Yang F, et al. Viral reactivation in the lungs of patients with severe pneumonia is associated with increased mortality, a multicenter, retrospective study. J Med Virol. 2023;95(1):e28337. doi: 10.1002/jmv.28337 36418241 PMC10099828

[2] Ramirez JA, Wiemken TL, Peyrani P, Arnold FW, Kelley R, Mattingly WA, et al. Adults hospitalized with pneumonia in the united states: incidence, epidemiology, and mortality. Clin Infect Dis. 2017;65(11):1806–12. doi: 10.1093/cid/cix647 29020164

[3] Torres A, Cilloniz C, Niederman MS, Menéndez R, Chalmers JD, Wunderink RG, van der Poll T, Pneumonia, Nature reviews. Disease primers.2021;7(1): 25. 33833230 10.1038/s41572-021-00259-0

[4] Cillóniz C, Torres A, Niederman MS. Management of pneumonia in critically ill patients. BMJ. 2021;375:e065871. doi: 10.1136/bmj-2021-065871 34872910

[5] GBD 2019 Diseases and Injuries Collaborators. Global burden of 369 diseases and injuries in 204 countries and territories, 1990-2019: a systematic analysis for the Global Burden of Disease Study 2019. Lancet. 2020;396(10258):1204–22. 33069326 10.1016/S0140-6736(20)30925-9 PMC7567026

[6] Xie J, Li Y, Wang M, He W, Zhao X. Diagnostic and Prognostic Value of Dysregulated miR-10a-3p in Patients with Severe Pneumonia. J Inflamm Res. 2022;15:6097–104. doi: 10.2147/JIR.S380818 36386576 PMC9645114

[7] Mo X, Jian W, Su Z, Chen M, Peng H, Peng P, et al. Abnormal pulmonary function in COVID-19 patients at time of hospital discharge. Eur Respir J. 2020;55(6):2001217. doi: 10.1183/13993003.01217-2020 32381497 PMC7236826

[8] Nan W, Li S, Wan J, Peng Z. Association of mean RDW values and changes in RDW with in-hospital mortality in ventilator-associated pneumonia (VAP): Evidence from MIMIC-IV database. Int J Lab Hematol. 2024;46(1):99–106. doi: 10.1111/ijlh.14192 37864327

[9] Xu C, Liu H, Zhang H, Zeng J, Li Q, Yi Y, et al. Predictive value of arterial blood lactate to serum albumin ratio for in-hospital mortality of patients with community-acquired pneumonia admitted to the Intensive Care Unit. Postgrad Med. 2023;135(3):273–82. doi: 10.1080/00325481.2022.2110769 35930266

[10] Li L, Zou G, Liu J. Preoperative glucose-to-lymphocyte ratio is an independent predictor for acute kidney injury after cardiac surgery in patients in intensive care unit. Int J Gen Med. 2021;14:6529–37. doi: 10.2147/IJGM.S335896 34675620 PMC8518472

[11] Hu T, Liu X, Liu Y. Usefulness of glucose to lymphocyte ratio to predict in-hospital mortality in patients with aecopd admitted to the intensive care unit. COPD. 2022;19(1):158–65. doi: 10.1080/15412555.2022.2052272 35392756

[12] Zhang Y, Zhang S. Prognostic value of glucose-to-lymphocyte ratio in critically ill patients with acute respiratory distress syndrome: a retrospective cohort study. J Clin Lab Anal. 2022;36(5):e24397. doi: 10.1002/jcla.24397 35358348 PMC9102764

[13] Yang S, Liu Y, Wang S, Cai Z, Yang A, Hui X. Association between high serum blood glucose lymphocyte ratio and all-cause mortality in non-traumatic cerebral hemorrhage: a retrospective analysis of the MIMIC-IV database. Front Endocrinol (Lausanne). 2023;14:1290176. doi: 10.3389/fendo.2023.1290176 38093959 PMC10718300

[14] Chen Y, Tang S, Wang Y. Prognostic value of glucose-to-lymphocyte ratio in critically Ill patients with acute pancreatitis. Int J Gen Med. 2021;14:5449–60. doi: 10.2147/IJGM.S327123 34526812 PMC8436258

[15] Liu J, Hu X. Association between glucose-to-lymphocyte ratio and in-hospital mortality in acute myocardial infarction patients. PLoS One. 2023;18(12):e0295602. doi: 10.1371/journal.pone.0295602 38060551 PMC10703328

[16] Johnson AEW, Bulgarelli L, Shen L, Gayles A, Shammout A, Horng S, et al. MIMIC-IV, a freely accessible electronic health record dataset. Sci Data. 2023;10(1):1. doi: 10.1038/s41597-022-01899-x 36596836 PMC9810617

[17] Pollard TJ, Johnson AEW, Raffa JD, Celi LA, Mark RG, Badawi O. The eICU Collaborative Research Database, a freely available multi-center database for critical care research. Sci Data. 2018;5:180178. doi: 10.1038/sdata.2018.178 30204154 PMC6132188

[18] Yang M, Zhang Q, Ge Y-Z, Tang M, Hu C-L, Wang Z-W, et al. Prognostic roles of glucose to lymphocyte ratio and modified glasgow prognosis score in patients with non-small cell lung cancer. Front Nutr. 2022;9:871301. doi: 10.3389/fnut.2022.871301 35619963 PMC9127733

[19] Zhang Y, Zheng S-P, Hou Y-F, Jie X-Y, Wang D, Da H-J, et al. A predictive model for frequent exacerbator phenotype of acute exacerbations of chronic obstructive pulmonary disease. J Thorac Dis. 2023;15(12):6502–14. doi: 10.21037/jtd-23-931 38249857 PMC10797373

[20] Paolisso P, Foà A, Bergamaschi L, Angeli F, Fabrizio M, Donati F, et al. Impact of admission hyperglycemia on short and long-term prognosis in acute myocardial infarction: MINOCA versus MIOCA. Cardiovasc Diabetol. 2021;20(1):192. doi: 10.1186/s12933-021-01384-6 34560876 PMC8464114

[21] Pan H, Xiong Y, Huang Y, Zhao J, Wan H. Association between stress hyperglycemia ratio with short-term and long-term mortality in critically ill patients with ischemic stroke. Acta Diabetol. 2024;61(7):859–68. doi: 10.1007/s00592-024-02259-4 38499778

[22] Iacobellis G, Penaherrera CA, Bermudez LE, Bernal Mizrachi E. Admission hyperglycemia and radiological findings of SARS-CoV2 in patients with and without diabetes. Diabetes Res Clin Pract. 2020;164:108185. doi: 10.1016/j.diabres.2020.108185 32360710 PMC7251996

[23] Gill SK, Hui K, Farne H, Garnett JP, Baines DL, Moore LSP, et al. Increased airway glucose increases airway bacterial load in hyperglycaemia. Sci Rep. 2016;6:27636. doi: 10.1038/srep27636 27273266 PMC4897689

[24] Torres A, Blasi F, Dartois N, Akova M. Which individuals are at increased risk of pneumococcal disease and why? Impact of COPD, asthma, smoking, diabetes, and/or chronic heart disease on community-acquired pneumonia and invasive pneumococcal disease. Thorax. 2015;70(10):984–9. doi: 10.1136/thoraxjnl-2015-206780 26219979 PMC4602259

[25] Saibal MAA, Rahman SHZ, Nishat L, Sikder NH, Begum SA, Islam MJ, et al. Community acquired pneumonia in diabetic and non-diabetic hospitalized patients: presentation, causative pathogens and outcome. Bangladesh Med Res Counc Bull. 2012;38(3):98–103. doi: 10.3329/bmrcb.v38i3.14336 23540185

[26] Kornum JB, Thomsen RW, Riis A, Lervang H-H, Schønheyder HC, Sørensen HT. Diabetes, glycemic control, and risk of hospitalization with pneumonia: a population-based case-control study. Diabetes Care. 2008;31(8):1541–5. doi: 10.2337/dc08-0138 18487479 PMC2494631

[27] McDonald HI, Nitsch D, Millett ERC, Sinclair A, Thomas SL. New estimates of the burden of acute community-acquired infections among older people with diabetes mellitus: a retrospective cohort study using linked electronic health records. Diabet Med. 2014;31(5):606–14. doi: 10.1111/dme.12384 24341529 PMC4264938

[28] Boyanova L, Mitov I. Antibiotic resistance rates in causative agents of infections in diabetic patients: rising concerns. Expert Rev Anti Infect Ther. 2013;11(4):411–20. doi: 10.1586/eri.13.19 23566150

[29] Di Yacovo S, Garcia-Vidal C, Viasus D, Adamuz J, Oriol I, Gili F, et al. Clinical features, etiology, and outcomes of community-acquired pneumonia in patients with diabetes mellitus. Medicine (Baltimore). 2013;92(1):42–50. doi: 10.1097/MD.0b013e31827f602a 23263718 PMC5370750

[30] Chen S, Hou C, Kang Y, Li D, Rong J, Li Z. Factors affecting hospital discharge outcomes in patients with community-acquired pneumonia: a retrospective epidemiological study (2014-2021). Am J Med Sci. 2023;366(2):143–9. doi: 10.1016/j.amjms.2023.05.008 37220846

[31] Liu D, Lan L, Luo D, Zhao B, Wei G, He Y, et al. Lymphocyte subsets with the lowest decline at baseline and the slow lowest rise during recovery in COVID-19 critical illness patients with diabetes mellitus. Diabetes Res Clin Pract. 2020;167:108341. doi: 10.1016/j.diabres.2020.108341 32707212 PMC7373679

[32] Shi X, LeCapitaine NJ, Rudner XL, Ruan S, Shellito JE. Lymphocyte apoptosis in murine Pneumocystis pneumonia. Respir Res. 2009;10(1):57. doi: 10.1186/1465-9921-10-57 19558669 PMC2714500

[33] Schreiber T, Swanson PE, Chang KC, Davis CC, Dunne WM, Karl IE, et al. Both gram-negative and gram-positive experimental pneumonia induce profound lymphocyte but not respiratory epithelial cell apoptosis. Shock. 2006;26(3):271–6. doi: 10.1097/01.shk0000225856.32260.0d 16912652

[34] Cao C, Yu M, Chai Y. Pathological alteration and therapeutic implications of sepsis-induced immune cell apoptosis. Cell Death Dis. 2019;10(10):782. doi: 10.1038/s41419-019-2015-1 31611560 PMC6791888

[35] Berbudi A, Rahmadika N, Tjahjadi AI, Ruslami R. Type 2 diabetes and its impact on the immune system. Curr Diabetes Rev. 2020;16(5):442–9. doi: 10.2174/1573399815666191024085838 31657690 PMC7475801

[36] Li Z, Xu S, Shi J, Zhang Y. Pneumocystis pneumonia in a patient with diabetes mellitus: A case report. Medicine (Baltimore). 2023;102(5):e32290. doi: 10.1097/MD.0000000000032290 36749248 PMC9901983

[37] Frydrych LM, Bian G, O’Lone DE, Ward PA, Delano MJ. Obesity and type 2 diabetes mellitus drive immune dysfunction, infection development, and sepsis mortality. J Leukoc Biol. 2018;104(3):525–34. doi: 10.1002/JLB.5VMR0118-021RR 30066958

[38] Zhang Y, Xu Y, Wang D, Kuang T, Wu W, Xu X, et al. Prognostic value of preoperative glucose to lymphocyte ratio in patients with resected pancreatic cancer. Int J Clin Oncol. 2021;26(1):135–44. doi: 10.1007/s10147-020-01782-y 32959232

[39] Yılmaz A, Şimşek M, Hannarici Z, Büyükbayram ME, Bilici M, Tekin SB. The importance of the glucose-to-lymphocyte ratio in patients with hepatocellular carcinoma treated with sorafenib. Future Oncol. 2021;17(33):4545–59. doi: 10.2217/fon-2021-0457 34431372

[40] Xiang L, Zhou TJ, Zhou LL, Luo J, Qin Z, You JZ, et al. Influenza a virus and Streptococcus pneumonia coinfection potentially promotes bacterial colonization and enhances B lymphocyte depression and reduction. J Biol Regul Homeost Agents. 2019;33(5):1437–49. doi: 10.23812/19-240-A 31637902

