# Supplementary material for: Impact of glucose-to-lymphocyte ratio on mortality in patients with pneumonia: A retrospective cohort study based on MIMIC-IV and eICU-CRD
Source: PLoS One. 2026 Jan 9;21(1):e0338579. doi: 10.1371/journal.pone.0338579 (PMC12788627; doi:10.1371/journal.pone.0338579)
Supplement: S2 Table — (PDF) [file pone.0338579.s002.pdf]

Table S1 Comparison of baseline features between groups stratified by GLR quartile of eICU-CRD cohort

Categories	Q1 (N=1602)	Q12 (N=1600)	Q13 (N=1602)	Q4 (N=1601)	Total (N=6405)	P-value
Demography						
Gender, n (%)						0.002
Female	799 (49.9%)	729 (45.6%)	712 (44.4%)	697 (43.5%)	2937 (45.9%)	
Male	803 (50.1%)	871 (54.4%)	890 (55.6%)	904 (56.5%)	3468 (54.1%)	
Age (year)	63.4(46,80.8)	66.9(51,82.8)	67.9(52.9,82.9)	68.2(54,82.4)	66.6(50.8,82.4)	<0.001
Weight (kg)	83(55.8,110.2)	84.1(54.3,113.9)	83.8(55.3,112.3)	84(55.1,112.9)	83.7(55.1,112.3)	0.666
Vital signs						
HR (times/min)	94.9(74.1,115.7)	95.6(74.4,116.8)	95.7(75.4,116)	96.6(76.3, 116.9)	95.7(75,116.4)	0.153
RR (times/min)	22.2(15,29.4)	22.8(15.8,29.8)	22.9(16,29.8)	23.5(16.2, 30.8)	22.9(15.8,30)	<0.001
Temperature (℃)	36.8(35.8,37.8)	36.9(35.9,37.9)	36.9(36,37.8)	36.9(36,37.8)	36.9(35.9,37.9)	0.016
SpO_2 (%)	96.4(92,100.8)	95.9(91.5,100.3)	96.1(91.8,100.4)	95.7(91.3, 100.1)	96(91.6,100.4)	<0.001
Comorbidities, n (%)						
Hypertension	333 (20.8%)	351 (21.9%)	326 (20.3%)	303 (18.9%)	1313 (20.5%)	0.206
Diabetes	103 (6.4%)	98 (6.1%)	94 (5.9%)	99 (6.2%)	394 (6.2%)	0.931
Heart Failure	284 (17.7%)	310 (19.4%)	290 (18.1%)	299 (18.7%)	1183 (18.5%)	0.649
Myocardial Infarction	85 (5.3%)	79 (4.9%)	82 (5.1%)	77 (4.8%)	323 (5%)	0.925
Cancer	89 (5.6%)	103 (6.4%)	110 (6.9%)	157(9.8%)	459 (7.2%)	<0.001
Stroke	38 (2.4%)	34 (2.1%)	37 (2.3%)	22 (1.4%)	131 (2%)	0.167
Atrial Fibrillation	216 (13.5%)	264 (16.5%)	243 (15.2%)	303 (18.9%)	1026 (16%)	<0.001
Scores system						
APS III	57.5(33.7,81.3)	56.7(33,80.4)	56.1(33.8,78.4)	57.3(34.5, 80.1)	56.9(33.7,80.1)	0.263
GCS	11.8(8.9,14.7)	12(9.1,14.9)	12.3(9.6,15)	12.4(9.7,15.1)	12.1(9.3,14.9)	<0.001
Laboratory tests						
WBC (K/UL)	11.7(5,18.4)	13.3(6.6,20)	13.9(7,20.8)	16.4(2.9,29.9)	13.8(4.7,14.1)	<0.001
RBC (m/uL)	3.8(3,4.6)	3.8(3.1,4.5)	3.7(3,4.7)	3.7(3,4.7)	3.7(3,4.7)	<0.001

Neutrophils (K/UL)	9.3(2.6,16)	10.2(3.5,16.9)	10.4(3.6,17.2)	10.6(2.8,18.4)	10.1(3.1,17.1)	<0.001
lymphocyte (K/UL)	3.1(2.9,3.3)	1.2(0.8,1.6)	0.8(0.3,1.1)	0.4(0.2,0.6)	1.4(1.2,1.6)	<0.001
PLT (K/μL)	237.3(125.2,349.4)	231.3(123.2,339.4)	214.5(111.3,317.7)	195.2(99.4,291)	219.6(113.1,326.1)	<0.001
HGB (g/dL)	11.2(9,13.4)	11(8.8,13.2)	11(8.8,13.2)	10.8(8.7,12.9)	11(8.8,13.2)	<0.001
RDW (K/μL)	15.8(13.3,18.3)	15.8(13.5,18.1)	15.8(13.4,18.2)	16(13.5,18.5)	15.8(13.4,18.2)	0.181
Na (mmol/L)	138.8(133.3,144.3)	138.4(132.9,143.9)	137.8(132.1,143.5)	137.4(132,142.8)	138.1(132.6,143.6)	<0.001
K (mmol/L)	4.1(3.5,4.7)	4.2(3.6,4.8)	4.2(3.6,4.8)	4.2(3.6,4.8)	4.2(3.6,4.8)	0.002
Ca (mg/dL)	8.4(7.6,9.2)	8.4(7.6,9.2)	8.4(7.6,9.2)	8.3(7.5,9.1)	8.3(7.5,9.1)	0.002
Glu (K/UL)	127.4(87,167.8)	143.2(99.3,187.1)	156.8(106.7,206.9)	181.1(120.2,242)	152.1(98.9,205.3)	<0.001
BUN (mg/dL)	27.9(7.6,48.2)	30.9(8.3,53.5)	32.5(10.9,54.1)	34.9(11.3,58.5)	31.5(9.3,53.7)	<0.001
Cr (mg/dL)	1.6(0,3.2)	1.6(0,3.2)	1.7(0.2,3.2)	1.7(0.1,3.3)	1.7(0.1,3.3)	0.304
Treatment						
Mechanical ventilation, n (%)	651 (40.6%)	609 (38.1%)	621 (38.8%)	603 (37.7%)	2484 (38.8%)	0.319
Vasoactive drugs,n (%)	294 (18.4%)	290 (18.1%)	312 (19.5%)	294 (18.4%)	1190 (18.6%)	0.759
Outcome						
In-hospital mortality, n (%)	219 (13.7%)	232 (14.5%)	278 (17.4%)	324 (20.2%)	1053 (16.4%)	<0.001
ICU mortality, n (%)	140 (8.7%)	137 (8.6%)	173 (10.8%)	202 (12.6%)	652 (10.2%)	<0.001

Table S2 Cox proportional hazard ratio for all-cause mortality in patients with bacterial pneumonia.

Categories		Model I HR (95 %CI)	P	Model II HR (95 %CI)	P	Model III HR (95 %CI)	P	Model IV HR (95 %CI)	P
In-hospital mortality	Q1	Ref.		Ref.		Ref.		Ref.	
	Q2	1.32 (0.64-2.72, p=.454)	0.454	1.06 (0.51-2.20)	0.868	1.06 (0.51-2.19)	0.871	1.02 (0.49-2.11)	0.965

	Q3	1.94 (1.02-3.69)	0.043	1.56 (0.81-2.97)	0.181	1.46 (0.76-2.81)	0.251	1.45 (0.76-2.79)	0.259
	Q4	3.33 (1.79-6.21)	＜0.001	2.75 (1.46-5.17)	0.002	2.76 (1.46-5.22)	0.002	2.65 (1.40-5.03)	0.003
ICU mortality	Q1	Ref.		Ref.		Ref.		Ref.	
	Q2	1.59 (0.61-4.10)	0.34	1.51 (0.58-3.90)	0.397	1.24 (0.47-3.24)	0.665	1.18 (0.44-3.16)	0.74
	Q3	1.99 (0.81-4.90)	0.133	2.08 (0.84-5.14)	0.112	1.77 (0.70-4.4)	0.226	1.59 (0.63-4.04)	0.329
	Q4	4.74 (2.02-11.13)	＜0.001	3.88 (1.61-9.31)	0.002	3.89 (1.61-9.40)	0.003	3.80 (1.56-9.29)	0.003
