# Supplementary material for: Impact of glucose-to-lymphocyte ratio on mortality in patients with pneumonia: A retrospective cohort study based on MIMIC-IV and eICU-CRD
Source: PLoS One. 2026 Jan 9;21(1):e0338579. doi: 10.1371/journal.pone.0338579 (PMC12788627; doi:10.1371/journal.pone.0338579)
Supplement: S3 Table — (PDF) [file pone.0338579.s003.pdf]

Table S2 Cox proportional hazard ratio for all-cause mortality in patients with bacterial pneumonia.

Categories		Model I HR (95 %CI)	P	Model II HR (95 %CI)	P	Model III HR (95 %CI)	P	Model IV HR (95 %CI)	P
In-hospital mortality	Q1	Ref.		Ref.		Ref.		Ref.	
	Q2	1.32 (0.64-2.72, p=.454)	0.454	1.06 (0.51-2.20)	0.868	1.06 (0.51-2.19)	0.871	1.02 (0.49-2.11)	0.965
	Q3	1.94 (1.02-3.69)	0.043	1.56 (0.81-2.97)	0.181	1.46 (0.76-2.81)	0.251	1.45 (0.76-2.79)	0.259
	Q4	3.33 (1.79-6.21)	< 0.001	2.75 (1.46-5.17)	0.002	2.76 (1.46-5.22)	0.002	2.65 (1.40-5.03)	0.003
ICU mortality	Q1	Ref.		Ref.		Ref.		Ref.	
	Q2	1.59 (0.61-4.10)	0.34	1.51 (0.58-3.90)	0.397	1.24 (0.47-3.24)	0.665	1.18 (0.44-3.16)	0.74
	Q3	1.99 (0.81-4.90)	0.133	2.08 (0.84-5.14)	0.112	1.77 (0.70-4.4)	0.226	1.59 (0.63-4.04)	0.329
	Q4	4.74 (2.02-11.13)	< 0.001	3.88 (1.61-9.31)	0.002	3.89 (1.61-9.40)	0.003	3.80 (1.56-9.29)	0.003
